# The impact of institutional repositories: a systematic review

**DOI:** 10.5195/jmla.2020.856

**Published:** 2020-04-01

**Authors:** Michelle R. Demetres, Diana Delgado, Drew N. Wright

**Affiliations:** Scholarly Communications Librarian, Samuel J. Wood Library, Weill Cornell Medicine, New York, NY, mrd2006@med.cornell.edu, http://orcid.org/0000-0002-4997-7707; Associate Director, Information, Education and Clinical Services, Samuel J. Wood Library & C.V. Starr Biomedical Information Center, Weill Cornell Medicine, New York, NY, did2005@med.cornell.edu, https://orcid.org/0000-0002-6290-3497; Research Librarian, Weill Cornell Medicine, New York, NY, drw2004@med.cornell.edu, https://orcid.org/0000-0002-1776-5427

## Abstract

**Objective:**

Institutional repositories are platforms for presenting and publicizing scholarly output that might not be suitable to publish in a peer-reviewed journal or that must meet open access requirements. However, there are many challenges associated with their launch and up-keep. The objective of this systematic review was to define the impacts of institutional repositories (IRs) on an academic institution, thus justifying their implementation and/or maintenance.

**Methods:**

A comprehensive literature search was performed in the following databases: Ovid MEDLINE, Ovid EMBASE, the Cochrane Library (Wiley), ERIC (ProQuest), Web of Science (Core Collection), Scopus (Elsevier), and Library, Information Science & Technology Abstracts (EBSCO). A total of 6,593 citations were screened against predefined inclusion and exclusion criteria.

**Results:**

Thirteen included studies were divided into 3 areas of impact: citation count, exposure or presence, and administrative impact. Those focusing on citation count (n=5) and exposure or presence (n=7) demonstrated positive impacts of IRs on institutions and researchers. One study focusing on administrative benefit demonstrated the utility of IRs in automated population of ORCID profiles.

**Conclusion:**

Based on the available literature, IRs appear to have a positive impact on citation count, exposure or presence, and administrative burden. To draw stronger conclusions, more and higher-quality studies are needed.

## INTRODUCTION

Gibbons defines an institutional repository (IR) as having the following core features: digital, community-driven and focused, institutionally supported, durable and permanent, and accessible [1]. These qualities make an IR an ideal platform for presenting and publicizing scholarly output that might not be suitable for publication in a peer-reviewed journal or that must meet open access (OA) requirements. This can include, but is not limited to, student work, presentations, working papers, conference papers, newsletters, electronic theses and dissertations (ETDs), journals with limited distribution, or electronic archival materials. In creating an OA platform to showcase an institution’s scholarly products, the benefits would seem to be self-evident, as the “OA advantage” in the traditional publishing environment has been widely discussed [2–4].

However, the challenges to developing an IR are varied and well documented [5–7]. Storage and staffing costs, low usage, faculty reticence to deposit in IRs, and time all align as reasons against the implementation and continued development of an IR at an academic institution. This systematic review aimed to define the various impacts that an IR can provide for an academic institution, thus justifying its implementation or maintenance.

## METHODS

This study was performed following the Preferred Reporting Items for Systematic Reviews and Meta-Analyses (PRISMA) statement [8]. In adherence to these guidelines, a protocol was registered in PROSPERO, an international prospective register of systematic reviews (registration # CRD42018091449).

### Search strategy

Medical librarians performed comprehensive literature searches to identify studies that evaluated the impact of IRs on academic institutions. Initial searches were run on April 27, 2018, with an updated search on March 4, 2019. The following databases were searched: Ovid MEDLINE (ALL: 1946 to present), Ovid EMBASE (1974 to present), the Cochrane Library (Wiley), ERIC (ProQuest), Web of Science (Core Collection), Scopus (Elsevier), and Library, Information Science & Technology Abstracts (EBSCO). Google Scholar was not searched because of its inherent lack of reproducibility and unclear indexing practices. Search terms included all subject headings and associated keywords for “institutional repository,” “open access publishing,” “pre-print repository,” or “academic repository.” Specific IR names, derived from OpenDOAR, were also used as search terms [9]. The full search strategy for Ovid MEDLINE is available in the supplemental appendix. There were no language, publication date, or article type restrictions on the search strategy.

### Study selection

After results were de-duplicated, 2 scholarly communications librarians independently screened a total of 6,593 citations using Covidence, a systematic review tool [10]. Discrepancies were resolved by consensus. Titles and abstracts were reviewed against predefined inclusion and exclusion criteria. Articles considered for inclusion were those that discussed demonstrated, measurable, or quantitative impacts of IRs. In light of the vast differences among repository types and content, the authors chose to focus only on IRs that were affiliated with academic institutions. For the purposes of this study, an academic institution was defined as an institution dedicated to education and research that grants academic degrees. An IR from an academic institution was defined as a web-based repository that exclusively supported content created by members of said academic institution. Excluded studies were those that focused on non-academic institutions or OA platforms that were not institutionally affiliated. Excluded studies also included those that only discussed assumed or eventual impacts or benefits.

**Figure 1:**
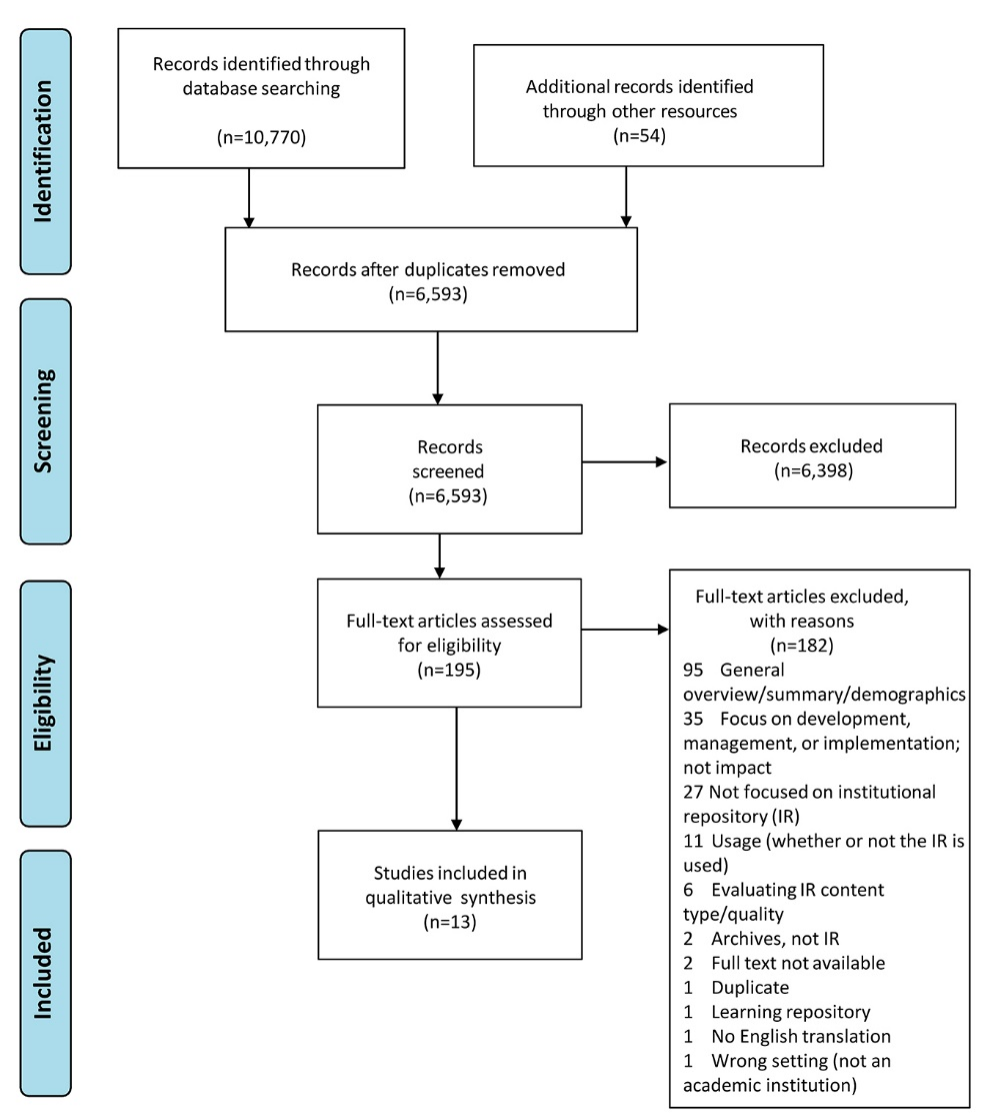
Preferred Reporting Items for Systematic Reviews and Meta-Analyses (PRISMA) flow diagram

Full text was then pulled for selected studies for a second round of eligibility screening. Reference lists and citing articles for the studies selected for inclusion were also pulled and searched. A total of thirteen studies were selected for inclusion in this review. Figure 1 shows the full PRISMA flow diagram outlining the study selection process.

### Data collection

Data from all included studies were pulled by two independent reviewers, assessing study design and outcomes, with results confirmed by consensus. After all included studies were reviewed, three areas of impact emerged: citation impact, exposure or presence, and administrative impact. Risk of bias was assessed at the individual study level according to standards set by the Cochrane Collaboration Qualitative Methods Group [11]. Per the standards, assessment of study quality included (i) adequacy of reporting detail, (ii) technical rigor and methodological soundness, and (iii) paradigmatic sufficiency. Due to the heterogeneity of the studies, no synthesis of results was performed.

## RESULTS

The thirteen studies were divided into three areas of impact: citation count, exposure or presence, and administrative impact. Table 1 describes all included studies.

**Table 1:**
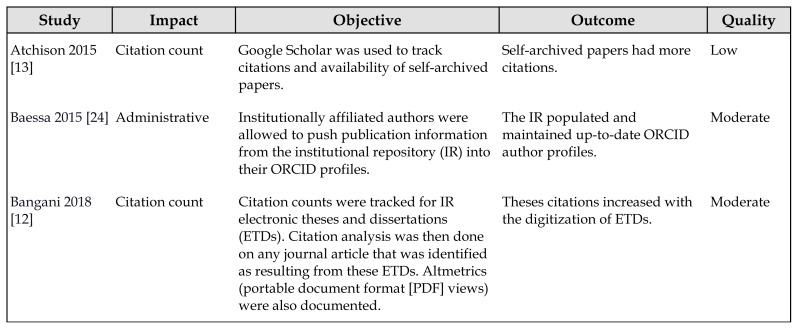
Included studies

Study	Impact	Objective	Outcome	Quality
Atchison 2015 [13]	Citation count	Google Scholar was used to track citations and availability of self-archived papers.	Self-archived papers had more citations.	Low
Baessa 2015 [24]	Administrative	Institutionally affiliated authors were allowed to push publication information from the institutional repository (IR) into their ORCID profiles.	The IR populated and maintained up-to-date ORCID author profiles.	Moderate
Bangani 2018 [12]	Citation count	Citation counts were tracked for IR electronic theses and dissertations (ETDs). Citation analysis was then done on any journal article that was identified as resulting from these ETDs. Altmetrics (portable document format [PDF] views) were also documented.	Theses citations increased with the digitization of ETDs.	Moderate
Bruns 2014 [17]	Exposure or presence	Download statistics for master’s theses were examined.	Thesis downloads from IR outpaced downloads from WorldCat.	Very low
Fan 2015 [18]	Exposure or presence	The contribution of IRs to their home institutions was calculated in terms of 4 webometric indicators: page counts, PDF counts, uniform resource locator (URL) mention counts, and link counts.	IRs improved webometric indicators of home institutions.	Low
Gargouri 2010 [14]	Citation count	Citation counts were compared between IR-deposited open access (OA) and non-OA articles published in the same (non-OA) journals.	OA due to deposition in IRs results in more citations.	Moderate
Linde 2012 [19]	Exposure or presence	The availability of conference proceedings stored in 6 IRs was examined.	25% of conference proceedings examined were only found in an IR.	Low
Organ 2006 [20]	Exposure or presence	Download statistics, page views, and cover views were tracked for an IR.	Materials in the IR were discoverable via Google more quickly than traditional publishing; downloads primarily came from Google.	Low
Pitol 2014 [15]	Citation count	Citation counts were collected via Google Scholar from an ~1,000-paper sample from 3 institutions.	Depositing in an IR, in combination with a listing in PubMed, resulted in more citations.	Low
Smith 2011 [21]	Exposure or presence	Internal links generated via Yahoo to IRs were traced back to Wikipedia.	Theses in IRs were used as evidence for Wikipedia articles.	Low
Smith 2013 [16]	Citation count	Deposit ratios of IRs with URL citation internal link counts were compared.	IRs with higher deposition rates were associated with more citations of their content.	Low
Stone 2014 [22]	Exposure or presence	Citations for ETDs in 49 IRs were tracked via Google Scholar.	ETDs were cited in peer-reviewed journals.	Low
Van Wyk 2014 [23]	Exposure or presence	Usage statistics of materials in IRs based on geographical location were evaluated.	IRs enhanced access to the global research community.	Low

### Citation count

Five of the thirteen included studies described the positive impact of IRs on citation count [12–16]. The most compelling of these, Gargouri 2010, demonstrated that the OA status of a paper resulting from its deposition in an IR was a statistically significant, independent, positive predictor of citation count, even when controlling for many other salient variables such as article age, journal impact factor, number of authors, number of pages, number of references cited, type of article, classification as a scientific article, and whether the first author was from the United States [14].

ETDs in IRs also appeared to benefit from an OA advantage. Bangani 2018 tracked citation counts obtained through Google Scholar for ETDs published from 1989 to 2014 from North-West University in South Africa and found a marked increase in citations after ETDs began to be digitized. A total of 612 ETDs had 931 citations, translating to 1.52 citations per ETD on average. Prior to digitization, however, a total of 81 theses and dissertations had only 10, translating to only 0.12 citations per document on average [12]. This positive citation impact pertained not only to the ETDs themselves, but also any published papers resulting from them. Pitol 2014 consistently found that IR deposition was the most impactful author-controlled way to make papers freely available in Google Scholar searches [15]. In combination with a listing in PubMed, IR deposition of papers resulted in significantly more citations than papers that did not have freely available full text that could be found via Google Scholar.

### Exposure or presence

Seven of the thirteen included studies focused on the increased exposure or discoverability that IRs provided for their content and their institutions [17–23]. This was measured in a variety of ways, including download statistics, webometric indicators (e.g., page views and internal links), and availability.

Linde 2012 saw increased exposure through the uniqueness of their collection: of the papers examined, 25% were not found digitally anywhere but the IR [19]. Van Wyk 2014 also spoke to the uniqueness of their collection at the University of Zululand, where exposure for their institutional output increased through the IR’s overseas usage, which accounted for 5% of its overall usage [23]. Organ 2006 discussed the speed with which the IR made discoverability possible. Of the 80.9% of downloads coming from Google, most materials were discoverable by Google within 24–48 hours [20]. Bruns 2014 saw that a thesis, after being made available in the IR for 1 year, was downloaded 729 times. Approximately a year after that, the thesis was downloaded well over 3,500 times. Prior to IR deposition, this same thesis had only been downloaded 35 times during a 1-year period [17].

### Administrative

Baessa 2015 discussed the unique administrative benefit provided by their IR [24]. The King Abdullah University of Science and Technology (KAUST) leveraged the benefit of the IR by allowing KAUST-affiliated authors to push publication information from the IR into their ORCID profiles. This helped authors maintain a current public profile without having to manually update their profiles themselves.

## DISCUSSION

One limitation of this review was that, for the most part, the quality of the articles discussing the impact of IRs was poor. Few discussed measurable or quantitative benefits, whereas many discussed the overall development of their IRs and their anticipated impact. Methodology was generally poor as well, with small sample sizes and questionable rationales. Therefore, more quantitative, methodologically rigorous studies are needed in this area.

The goal of making underrepresented work, such as ETDs, discoverable and citable can be achieved through making this work available in an IR and discoverable by Google and Google Scholar, which is also often linked to an improved citation rate. As Stone 2014 points out, “many senior theses are about regional and local issues or cutting-edge topics, both of which may have a dearth of publications in the mainstream scholarly literature due to interest and/or the scholarly publishing cycle” [22]. This makes an IR an important source for studies on new or niche topics and an accessible avenue for scholars.

Indexing and discoverability are the main reasons for the difference in the “OA advantage” (e.g., more citations) between IRs and OA journals. OA journals are often widely indexed in databases like MEDLINE, EMBASE, and Scopus, which makes discoverability for OA journal articles, and therefore citations, much more likely. IRs are typically only discoverable through Google and Google Scholar or OA content aggregators such as CORE [25]. The Institutional Repository LinkOut feature in PubMed is still not widely adopted, with only 36 IRs currently participating [26]. This is likely due to the difficult requirements for IR application and acceptance (e.g., minimum of 1,000 articles that are not already deposited in PubMed Central) [27].

However, the literature suggests that IRs can still be an important outlet for exposure, particularly when used for preprints and data. Conroy found “journal articles that were uploaded as preprints before being published gather more citations in the long run than papers without a preprint version” [28]. The effect is similar with regard to the availability of data. One study found that articles with data availability statements have up to 25% higher citation impact on average [29]. Another found that publicly available data are associated with a “69% increase in citations, independently of journal impact factor, date of publication, and author country of origin” [30]. Most articles included in this review focused on ETDs or traditional journal articles. However, IRs are also important potential platforms for the exposure and citation of preprints and data.

One justification for IRs comes from the Linde 2012 study, which reports that 25% of conference proceedings examined were only found in the IR [19]. The inaccessibility of conference or meeting abstracts is a problem frequently confronting librarians and researchers, especially those who seek to include grey literature in systematic reviews. Not all conferences publish proceedings, effectively making this work inaccessible. For those that do, the representation of the work is often incomplete or impermanent. For example, poster presentations are frequently published as written abstracts only, omitting the context and graphics that would be included in a physical poster. This is not the case with IRs, in which complete posters can be made available.

Moreover, conference and meeting abstracts are usually published as supplements, for which online access is not always guaranteed in perpetuity. Finding full text for older abstracts is often impossible. If published on association websites, the abstracts may be made available only to attendees of the specific meeting or members of the organization [31]. Furthermore, conference and meeting abstracts often do not result in full-length publications [32–34], with one study showing that of a total of 29,729 abstracts presented at scientific meetings, the rate of weighted mean abstracts that were fully published was 44.5% [35]. This suggests that the conference and meeting abstracts are the only source of a large portion of scientific information. Together, these reasons make a compelling argument for using IRs as outlets for conference and meeting abstracts and posters.

The studies included in this review also provide evidence of the global impact of IRs. Six of the thirteen included studies came from institutions outside the United States [12, 18–20, 23, 24]. This speaks to the interconnectivity that IRs can facilitate, making scholarship available internationally. Much discussion in scholarly communication circles focuses on the disparity in access to and production of research between the Global North and Global South [36–38]. As Vattikoti points out, “Most of the countries in Global south are not in a position to afford such huge fees charged by pay-access publishers because of insufficient funds or prioritization of the limited research funds for carrying out research activities” [39]. By contributing to a free-to-deposit OA platform like an IR, researchers help bridge this scholarship gap and position IRs as an important resource in equitizing academic scholarship.

Based on the available literature, the authors found that IRs appear to have a positive impact on citation count, exposure or presence, and administrative burden. To make stronger conclusions, more and higher-quality studies are needed.

## SUPPLEMENTAL FILE

Appendix The full search strategy for Ovid MEDLINE

## 

**Michelle R. Demetres**, mrd2006@med.cornell.edu, http://orcid.org/0000-0002-4997-7707, Scholarly Communications Librarian, Samuel J. Wood Library, Weill Cornell Medicine, New York, NY

**Diana Delgado, AHIP**, did2005@med.cornell.edu, https://orcid.org/0000-0002-6290-3497, Associate Director, Information, Education and Clinical Services, Samuel J. Wood Library & C.V. Starr Biomedical Information Center, Weill Cornell Medicine, New York, NY

**Drew N. Wright**, drw2004@med.cornell.edu, https://orcid.org/0000-0002-1776-5427, Research Librarian, Weill Cornell Medicine, New York, NY
