# Supplementary material for: The impact of institutional repositories: a systematic review
Source: J Med Libr Assoc. 2020 Apr 1;108(2):177–84. doi: 10.5195/jmla.2020.856 (PMC7069834; doi:10.5195/jmla.2020.856)
Supplement: Appendix [file jmla-108-177-s001.pdf]

JMLA Journal of the Medical Library Association

SC
Supplemental content to
J Med Libr Assoc. Apr;108(2):dx.doi.org/10.5195/jmla.2020.856
www.jmla.mlanet.org
© Demetres, Delgado, Wright 2020

The impact of institutional repositories: a systematic review

Michelle R. Demetres; Diana Delgado, AHIP; Drew N. Wright

APPENDIX

The full search strategy for Ovid MEDLINE

Ovid MEDLINE (ALL: 1946 to Present)

Searched on April 27, 2018; updated on March 4, 2019

No publication date, language, or article type limits

1 **Open Access Publishing/**

2 (open access publishing or open access platform).mp

3 (open access repository or open access repositories or institutional repository or institutional repositories or digital repository or digital repositories or university repository or university repositories or college repository or college repositories or research repository or research repositories or academic repository or academic repositories or preprint archive or preprint archives or pre-print archive or pre-print archives or preprint repository or preprint repositories or pre-print repository or pre-print repositories).mp.

4 (DSpace* or CONTENTdm or Digibib or Digi-Tool or Digital Commons* or DigitalCommons* or Diva-Portal or dLib or Drupl or EPrint* or ETD-db or Fedora or invenio or islandora or MyCore or Omeka or WEKO or XooNIps).mp

5 ("Ergani - Historical Archive of Aegean" or "4TU.Centre for Research Data" or "Aaltodoc Publication Archive" or "Aberdeen University Research Archive" or "Abertay Research Portal" or "ABU Zaria Research Publications" or "Academic Digital Library (Akademickiej Bibliotece Cyfrowej)" or "Academica-e" or "Access to Research and Communications Annals" or "Access to Research at National University of Ireland, Galway" or "Access to Research Resources for Teachers" or "ACEReSearch" or "Acervo Digital da Unesp" or "Ackerman Archives" or "ACKU Library Catalogue" or "ACMAC" or "Acropolis Educational Resources Repository" or "Acta Fracturae conference archive" or "Activos Digitales IAPH" or "ACU Research Bank" or "ACUBO (Archivio Aperto di Ateneo)" or "Addis Ababa University Libraries Electronic Thesis and Dissertations Database" or "Adelaide Research" or "Afganistan Digital Library" or "African Higher Education Research Online" or "Agatange" or "Agder University Research Archive" or "AgEcon Search" or "Agentlink Clearinghouse" or "AGI Repository" or "Agritrop" or "AHKRC Digital Library" or "AHS Memory Project" or "Ajaums Repository" or "Ajou Open Repository" or "Akdeniz Universitesi Kurumsal Arsivi" or "Alexandria Research Platform" or "Alfred University Research and Archives" or "ALICIA: Repositorio Nacional Digital de Ciencia" or "All Ireland Public Health Repository" or "Allen Park Veterans Administration Hospital Archives" or "Almae Matris Studiorum Campus" or "Altai State University Electronic library" or "American College of Healthcare Sciences, Theses and Capstone Projects" or "American Memory" or "American Mineralogist Crystal Structure Database" or "American Museum of Natural History Scientific Publications" or "AMS Acta" or "AMS Tesi di Dottorato" or "AMS Tesi di Laurea" or "AMU Repository (Knowledge Repository)" or "Anglia Ruskin Research Online" or "Ankara University Archive System" or "Ansenuza" or "ANSTO Publications Online" or "ANU Digital Collections" or "Aomori University of Heatlh and Welfare Repository A-

© Demetres, Delgado, Wright 2020

plus" or "Apeiron - IULM" or "Aphasiology Archive" or "Arhiivikeskuse digiarhiiv" or "Aristotle University of Thessaloniki Repository - Psifiothiki" or "Armenian Foundation Digital Library" or "Armenian Rare Books 1512-1800" or "ARMIDA@UniMi" or "ARROW@DIT" or "ART-Dok" or "ARTHRA" or "ARTUR-FC" or "arXiv" or "Asia University Academic Repositories" or "Silesia Digital Library" or "Association for the Scientific Study of Consciousness ePrints Archive" or "Aston Data Explorer" or "Aston Publications Explorer" or "Athenaeum@UGA" or "Atlantic Canada Virtual Archives" or "Auburn University Scholarly Repository" or "AUC DAR Repository" or "AUCA Open Electronic Library" or "AUE Repository" or "AUK Repository" or "Australian Digital Theses Program (ADT) - Australian Catholic University" or "AUT Scholarly Commons" or "B-Digital" or "B-Salut" or "Baku Higher Oil School Repository" or "Ball State University Digital Media Repository" or "BAM-Publica" or "Banco Internacional de Objetos Educacionais" or "Batman Universitesi Akademik Arsiv Sistemi" or "Bayerische StaatsBibliothek" or "Baylor Electronically Accessible Research Documents" or "BCU Open Access" or "BEAR (Buckingham E-Archive of Research)" or "Belarusian State University of Informatics and Radioelectronics Repository" or "Benguet State University Digital Library" or "Beppu University and Oita Regional Society Co-Operation" or "Beppu University Information Library for Documentation" or "bepress Legal Repository" or "Bergen Open Research Archive - CMI").mp.

6 ("Bergen Open Research Archive - UiB" or "Bergen Open Research Archive at Bergen University College" or "Berlin Brandenburgischen Akademie der Wissenschaften Dokumentenserver" or "Berman Jewish Policy Archive @ NYU Wagner" or "Bern Open Repository and Information System (BORIS)" or "BG Research Online" or "BI Open Archive" or "Biblio at Institute of Formal and Applied Linguistics" or "BiblioDigital SIDRE" or "Digital Library of Cracow University of Technology" or "Biblioteka Humanistyczna (Humanist Library)" or "Biblioteka Uniwersytetu Warszawskiego" or "Biblioteki Cyfrowej Politechniki Warszawskiej" or "Biblos-e Archivo" or "Bibloteca para la Persona (Library for the Person)" or "Bichler & Nitzan Archives" or "BICTEL/e" or "BICTEL/e - ULg" or "BieColl - Bielefeld Electronic Collections" or "Biens Culturels Africains" or "Bilecik University" or "Biodiversity Heritage Library OAI Repository" or "Biological Magnetic Resonance Data Bank" or "bioRxiv" or "Bioversity International Publications" or "Birkbeck Institutional Research Online" or "Black Abolitionist Archive" or "BLD - Bilboko Liburutegi Digitala" or "BOA - Bicocca Open Archive" or "Bochum Research Bibliography" or "Boise State University - ScholarWorks" or "Bolivarium" or "Boston College University Libraries Digital Collections" or "Botanicus Digital Library" or "Bouira University Digital Space" or "Bournemouth University Research Online" or "Bozok Univeristy Open Archive" or "Bradford Scholars" or "Brage - NIH" or "Brage HiM" or "Brage IMR" or "Brage INN" or "Brage NMBU" or "Brage Nord Open Research Archive" or "Brage NP" or "Brandeis University Digital Collections" or "Brasiliana USP" or "Bridge of the Nineteenth Century" or "British Columbia's network of post-secondary digital repositories" or "British History Online" or "Brunel University Research Archive" or "BSU Digital Library" or "Budapest Business School Repository (BORY)" or "BULERIA" or "Bulgarian Digital Mathematics Library at IMI-BAS" or "Bulgarian OpenAIRE Repository" or "Bushehr University of Medical Sciences Repository" or "BYU ScholarsArchive" or "Caerdydd" or "Calhoun, Institutional Archive of the Naval Postgraduate School" or "Caltech Archives Oral Histories Online" or "Caltech Authors" or "CalTech Engineering and Science Online" or "Caltech Theses and Dissertations" or "CaltechCONF" or "CamEPrints" or "CamPuce" or "Canadian Breast Cancer Research Alliance Open Access Archive" or "CancerData" or "Canterbury Research and Theses Environment" or "Cardinal Scholar" or "CARE - CCLHD Archive and Research E-Library" or "Carlyle Letters Online: A Victorian Cultural Reference" or "Carpe dIEN" or "CaSA NaRA" or "Catalogo dei prodotti della ricerca" or "CBPF Index" or "CDC Stacks" or "CEACS Repository" or "CEDA Repository" or "Celebration of Women Writers" or "CemOA" or "Center for Jewish History

SC
Supplemental content to
J Med Libr Assoc. Apr;108(2):dx.doi.org/10.5195/jmla.2020.856
www.jmla.mlanet.org
© Demetres, Delgado, Wright 2020

Digital Collections" or "Center of Academic Publications" or "Central and Eastern European Marine Repository" or "Central Archive at the University of Reading" or "Central Connecticut State University Digital Collections" or "Central Economics and Mathematics Institute RAS" or "Central Environmental Authority Repository" or "Central European University's Academic Archive" or "Central Florida Memory" or "Central Lancashire Online Knowledge" or "Central Michigan University's Online Digital Object Repository (CONDOR)" or "Central Research and Creativity Online" or "Central Ukrainian National Technical University Repozitory" or "CENTRALESUPELEC" or "Centre for European Reforms Studies" or "Cerist Digital Library" or "CERN Document Server" or "CeSGO").mp. ("CEU Repositorio Institucional" or "CGIAR Library" or "Chalmers Publication

7 Library" or "ChesterRep" or "ChinaXiv" or "Chopin Early Editions" or "CHSPR Publications" or "Chubu University Digital Archives" or "Chung Hwa University of Medical Technology Repository" or "CIAT Document Repository" or "CIAT Research Online" or "CICERO Research Archive" or "CiencIPCA" or "City Research Online" or "Claremont Colleges Digital Library" or "CLARIN service center of the Zentrum Sprache at the BBAW" or "Clearing House Transport" or "Clinical Medicine NetPrints" or "Coconut Research Institute Repository" or "Codices Electronici Sangallenses" or "Coleccion de Tesis Digitales -UAEH-" or "Colecciones Digitales Uniminuto" or "Collection Of Biostatistics Research Archive" or "Collections de Corpus Oraux Numeriques (COCOON)" or "Collections Online" or "Collegio Carlo Alberto Publications Repository" or "Cologne Open Science - Wissenschaft weltweit vernetzen" or "Colorado Plateau Digital Archives" or "Colpos digital" or "Columbia University Academic Commons" or "Combined Arms Research Library Digital Library" or "Comenius-Institut" or "Comillas" or "CommonKnowledge" or "Commonrepo UM" or "Community Repository of Fukui" or "Computer Laboratory Technical Reports - Cambridge University" or "computer science publication server" or "Computer Science Technical Reports @Virginia Tech" or "COMU Open Access System" or "CONICET Digital" or "Consortium for the Advancement of Undergraduate Statistics Education" or "COPELABS Scientific commons" or "Copenhagen University Research Information System" or "Cor-Ciencia" or "Cork Open Research Archive" or "Cornerstone: A Collection of Scholarly and Creative Works" or "Corpus Innovationis Europaensis" or "CorpusUL" or "CORUS" or "Corvinus Research Archive" or "Corvinus University of Budapest" or "Council of Independent Colleges Historic Campus Architecture Project" or "Covenant University Electronic Theses and Dissertation Repository" or "CPUT Electronic Theses and Dissertations Repository" or "Cranfield CERES" or "Creative Repository of Electro-Communications" or "CREST Repository" or "CRIS UNS" or "Cronfa at Swansea University" or "Cross Collection Discovery" or "CrossAsia-Repository" or "CRS4 Open Archive" or "CRTI Digital Library" or "Crystallography Open Database" or "CSIR Research Space" or "CSIRO Data Access Portal" or "CSIRO Research Publications Repository" or "CSU Research Output" or "CSUN ScholarWorks" or "CSUSB ScholarWorks" or "CUAL Repository (Connacht Ulster Alliance Libraries)" or "Culturally Authentic Pictorial Lexicon" or "CumInCAD Digital Archive" or "CUNY Academic Works" or "CurateND" or "Current Research Information System, TEI of Epirus" or "Carleton University Research Virtual Envronment" or "CyberLeninka - Russian open access scientific library" or "Cybertesis UNI" or "Cybertesis UNMSM" or "Czech digital mathematics library" or "d-Commons" or "D-Scholarship@Pitt" or "DAFWA Research Library" or "Dagstuhl Research Online Publication Server" or "Dalarna University College Electronic Archive" or "Dalhousie Computer Science Technical Reports Series" or "DalSpace" or "Datadoi" or "Dataspace" or "datorium" or "DBS Esource" or "DCU Online Research Access Service" or "DDFV" or "De Montfort University Open Research Archive" or "Deakin Research Online" or "Deep Blue at the University of Michigan" or "DeepBlue Knowledge Repository@PDPU" or "Defense Technical Information Centre").mp.

8 ("DENISON Digital Resource Commons" or "Department of Computer Science E-Repository" or

JMLA Journal of the Medical Library Association
SC
Supplemental content to
J Med Libr Assoc. Apr;108(2):dx.doi.org/10.5195/jmla.2020.856
www.jmla.mlanet.org
© Demetres, Delgado, Wright 2020

"Department of Computer Science Publications Archive" or "deposit::hagen" or "DepositOnce" or "Desarrolla, Aprende y Reutiliz" or "Desert Island Discs: Castaway Archive and Podcasts" or "DESY Publication Database" or "Detroit Public Library Digital Collections" or "Deutsche Sporthochschule K√Éln" or "DI-fusion" or "DIA Brage" or "DIALNET" or "Dicle University" or "Difunde" or "DigiBESS" or "Digilib UIN Sunan Kalijaga" or "DIGIMOM" or "DigiNole" or "Digitaalne Arhiiv" or "Digital Access to Scholarship at Harvard" or "Digital archive library of the MSU named after A.A. Kuleshov Instutional Repository" or "Digital Archive of Georgian Library Association" or "Digital Archives of Colorado College" or "Digital Archives of Literacy Narratives" or "Digital Assets Repository" or "Digital Collections - USC" or "Digital Collections @ FAU Libraries" or "Digital Collections Repository" or "Digital Collections@KUMC" or "Digital Collections@UM" or "Digital DU" or "Digital Education Resource Archive" or "Digital Himalaya" or "Digital Howard" or "Digital Innovation South Africa" or "Digital Karnak" or "Digital Knowledge at Cape Peninsula University of Technology" or "Digital Knowledge Repository of Central Drug Research Institute" or "Digital Land of Sieradz" or "Digital library (repository) of Tomsk State University" or "Digital Library Belarusian State Economic University" or "Digital library for Ardabil University of Medical Sciences" or "Digital Library for Earth System Education" or "Digital Library Jose Maria Vargas Vila" or "Digital Library NAPS of Ukraine" or "Digital Library of Book Studies" or "Digital library of Brno University of Technology" or "Digital Library of Chelm" or "Digital Library of Macedonia" or "Digital Library of Modern Greek Studies" or "Digital Library of Open University of Tanzania" or "Digital Library of Opole" or "Digital Library of Polish and Poland-Related News Pamphlets" or "Digital Library of Polish Institute of Anthropology" or "Digital Library of Polotsk State University" or "Digital Library of Slovenia" or "Digital Library of the Commons" or "Digital Library of the Czech Technical University in Prague" or "Digital Library of the Faculty of Arts, Masaryk University" or "Digital Library of The KARTA Center Foundation" or "Digital Library of the Tanzania Health Community" or "Digital Library of the University of Pardubice" or "Digital Library of UIN Sunan Ampel" or "Digital library of University of Maribor" or "Digital Library University of Lodz" or "Digital Mechanism and Gear Library" or "Digital Online Collection of Knowledge and Scholarship" or "Digital Online Repository and Information System" or "Digital Scholarship @ Tennessee State University" or "Digital South Asia Library" or "Digital Theses - University of Technology, Sydney" or "Digital Treasures Repository: Central/Western Massachusetts Resource Sharing Project" or "Digital UNC" or "Digital University Library Saxony-Anhalt" or "Digital USD" or "Digital Window @Vassar" or "Digital.CSIC" or "Digital.Maag" or "Digitala Vetenskapliga Arkivet - Academic Archive On-line" or "Digitale Bibliothek Braunschweig" or "Digitale Hochschulschriften der LMU" or "Digitale utgivelser ved UiO" or "DigitalLibrary@CUSAT" or "Digitalna zbirka Hrvatske akademije znanosti i umjetnosti" or "Digitalni arhiv Filozofskog fakulteta u Zagrebu" or "DigitalResearch@Fordham" or "Digitised Diseases" or "Digitized Rare Collection" or "DigitUnito" or "Digtial Repository of Red Sea University" or "Digtial Repository of White Nile University" or "Dipartimento di Fisica e Astronomia - UNICT" or "Diposit Digital de Documents de la UAB" or "Diposit Digital de la Universitat de Barcelona" or "DIR@IMTECH" or "DiscoverArchive" or "Dissertationen online" or "DLR publication server" or "DLynx - Rhodes College Archives Digital Collection").mp.

9 ("DMMH Brage" or "DOCS@RWU" or "DocTA - Doctoral Theses Archive" or "Document Server@UHasselt" or "Documentacion cientifica de la ULPGC en abierto" or "Documenting the American South" or "Documents Collection Centre" or "doiSerbia PhD" or "DoKS@ Katholieke Hogeschool, Limburg" or "Dokumentenrepositorium der RUB / RUB-Repository" or "Dokumentenserver der Akademie der Wissenschaften" or "Dokumentenserver der Georg-August" or "Dokumentenserver des LBI-HTA" or "Dokumentenserver Klimawandel" or "Dokumtenserver der Freien Berlin" or "Dokuz Eylul University Open Archive System" or "Dominican Scholar" or "DoPuS

Dokumenten- und Publikationsserver Speyer" or "DR-NTU (Data)" or "Drexel Libraries E-Repository and Archives" or "DRS at National Institute Of Oceanography" or "Dryad" or "DSTO Scientific Publications Online Repository" or "DUGiFonsEspecials - Universitat de Girona" or "Duisburg-Essen Publications Online" or "Duke Law Scholarship Repository" or "DukeSpace" or "Durham e-Theses" or "Durham Research Online" or "DUT IR" or "Duzce University Open Access" or "E Knowledge Center" or "e-ANAQUEL" or "e-artexte" or "e-cienciaDatos" or "e-Learning Repository" or "E-LIB Dokumentserver - Staats und Universit√É¬§tsbibliothek Bremen" or "E-Library on Disaster Management" or "E-LIS" or "E-Locus" or "E-Prints Complutense" or "e-Prints Soton" or "e-Publications@khm" or "e-publications@RCSI" or "e-publications@UNE" or "E-resource repository of the University of Latvia" or "e-Scholar@UOIT" or "e-space at Manchester Metropolitan University" or "E-theses of the University of Tuzla (PHAIDRA)" or "E-thesis - Electronic Publications at the University of Helsinki" or "eaDNURT - the electronic archive of the Dnepropetrovsk National University of Railway Transport" or "eArchives" or "eArsiv@Anadolu" or "earsiv@Yasar" or "Earth Simulator Research Results Repository" or "Earth-prints Repository" or "Eastern University Digital Library" or "eBangor" or "EBOOKS Repository" or "ECNIS-NIOM Repository (Environmental Cancer Risk, Nutrition and Individual Susceptibility)" or "ECOLIB-Library of National Research and Development Institute for Industrial Ecology" or "Ecological Restoration Institute - Northern Arizona University" or "eCommons@AKU" or "eCommons@Cornell" or "eCommons@USASK" or "Economics Network Online Learning and Teaching Materials" or "EconPort" or "EconStor" or "eCrystals - Southampton" or "Edge Hill Research Archive" or "Edinburgh DataShare" or "Edinburgh Research Archive" or "Edinburgh Research Explorer" or "eDiss" or "edoc" or "eDoc.VifaPol" or "edocUR" or "EdShare" or "Education and Research Archive" or "Education by Design The Bienes Center's WPA Museum Extension Project Collection" or "Educational Repository - University of Patras" or "EduDoc" or "EEPIS Repository" or "eGyankosh" or "EKE Repository of PhD Dissertations" or "EKE Repository of Publications" or "El Abrigo" or "Elar at YarSU" or "ELECTRA - Electronic Archive of the Institute for Regional Studies, Centre for Economic and Regional Studies, Hungarian Academy of Sciences" or "Electronic Archive Donetsk National Technical University" or "Electronic Archive Khmelnitskiy National University" or "Electronic Archive Odessa National Academy of Food Technologies" or "Electronic Archive of Kharkiv National Automobile and Highway University" or "Electronic Archive of Kharkov National University of Radioelectronics" or "Electronic Archive of Kyiv Polytechnic Institute" or "Electronic archive of Ternopil National Ivan Puluj Technical University" or "Electronic archive of the Ural State Forest Engineering University" or "Electronic archive of Tomsk Polytechnic University" or "Electronic Archive of Ukrainian Engineering Pedagogics Academy" or "Electronic archive USPU").mp.

10 ("Electronic arhive of Vitebsk State Medical University Library" or "Electronic Environmental Resources Library" or "Electronic Gateway for Icelandic Literature" or "Electronic Library Belinsky" or "Electronic library of Belarusian State Technological University" or "Electronic library of Belarusian Trade and Economics University of Consumer Cooperatives " or "Electronic library of Belarusian-Russian University" or "Electronic Library of Ukraine Open Archive " or "Electronic Library Pavel Sukhoi State Technical University of Gomel (GSTU)" or "Electronic photo archive of the Belarusian State University" or "Electronic Publication Information Center" or "Electronic Repository - Central Medical Library - MU, Sofia" or "Electronic Repository Kyiv National University of Technologies and Design" or "Electronic Repository of the National Pedagogical Dragomanov University" or "Electronic Theses and Dissertations at Indian Institute of Science" or "Electronic Thesis & Dissertations - Brigham Young University" or "Electronic Thesis and Dissertation Library - LSU" or "Elektronisch archivierte Theorie - Sammelpunkt" or "Elektronische Dokumente der EUV - OPUS" or "eLibrary National Mining University" or "ELOGeo Repository" or "Elpub digital library" or "EMBL-EBI's

Supplemental content to
J Med Libr Assoc. Apr;108(2):dx.doi.org/10.5195/jmla.2020.856
www.jmla.mlanet.org
© Demetres, Delgado, Wright 2020

Protein Data Bank in Europe (PDBe)" or "ENEA Open Archive" or "EngagedScholarship@CSU" or "English Heritage ViewFinder" or "eNGSUIR" or "ePLUS" or "Epsilon Archive for Student Projects" or "Epsilon Open Archive" or "Epsilon Undergraduate Theses Archive" or "EPub Bayreuth" or "ePublications" or "ePublications@Bond University" or "epublications@Marquette" or "ePublications@SCU" or "ePubs: the open archive for STFC research publications" or "Erciyes University Open Archive System" or "ERES Digital Library" or "eResearch@Ozyegin" or "ErUCU: Electronic repository of the Ukrainian Catholic University" or "ESC publication" or "eScholarShare at Drake University" or "eScholarship - University of California" or "eScholarship@BC" or "eScholarship@McGill" or "eScholarship@UMMS" or "eScriptorium" or "Escuela de Postgrado - Tesis Postgraduales" or "Escuela Superior Politecnica del Litoral" or "Esculela Politecnica Nacional Biblioteca Central: Tesis a texto completo (Ecuador)" or "ESE - Salento University Publishing" or "ESPACE ENAP" or "Espace INRS" or "espace@Curtin" or "ESRC Research Catalogue" or "ESTIA" or "Estudo Geral" or "ETD - Unsyiah Central Library" or "ETDs @ VT" or "Etheses - A Saurashtra University Library Service" or "Etheses of Maulana Malik Ibrahim State Islamic University" or "ethesis@nitr" or "Ethics in Science and Engineering National Clearinghouse" or "ETSU Electronic Thesis and Dissertation Archive" or "EURECOM Repository" or "Europe PubMed Central" or "European Centre for Minority Issues" or "European Cultural Heritage Online" or "European Documentation Centers" or "european film gateway" or "European Research Papers Archive" or "europeana 1914-1918" or "Euskal Doktorego Tesien Bilduma - Repositorio de Tesis Doctorales" or "Euskal Memoria Digitala" or "eVols at University of Hawaii at Manoa" or "EWU Digital Library" or "Explore Bristol Research" or "Fachlicher Dokumentenserver Paedagogik/Erziehungswissenschaften" or "Fachrepositorium Lebenswissenschaften - Life Sciences Repository" or "Faculty of Mining, Geology and Petroleum Engineering Repository" or "Faculty of Science Repository" or "Fagarkivet" or "FAMENA PhD Collection" or "FAMENA Repository" or "FAOBIB" or "FEDERAL UNIVERSITY NDUFU-ALIKE IKWO REPOSITORY ARCHIVE" or "Federal University Oye Ekiti Repository" or "FedUni Research Online" or "FHL Virtual Libraries" or "FHS Brage" or "FIDES Digital Library" or "FigShare" or "FIKT Repository" or "First Nations Digital Document Source" or "First World War Poetry Digital Archive" or "Fisher Digital Publications" or "Fiskeridirektoratets digitalarkiv" or "FJSL - Fondation Jeunes Scientifiques Luxembourg" or "FlacsoAndes").mp.

11 ("FLASH: The Fordham Law Archive of Scholarship and History" or "Flinders Academic Commons" or "Fluorophores.org" or "FM Repository" or "Folkstreams" or "Fontys iPort" or "Foodbase" or "Forced Migration Online Digital Library" or "FortWorks" or "FOSS Repository" or "FOundation LIterature Online" or "FPSZG repository" or "Fr. Edward J. Dowling, S.J. Marine Historical Collection" or "Fraunhofer-ePrints" or "Freiburger Dokumentenserver" or "FREIburger Multimedia Object Repository" or "FreiMusic - HfM Freiburg" or "Fundacion Mapfre" or "Funes" or "Furman University Scholar Exchange (FUSE)" or "Future University Hakodate Academic Archive" or "FWF-E-Book-Library" or "Galiciana" or "Gallica, Bibliotheque Numerique" or "GAMS - Asset Management System for the Humanities" or "Gateway to Oklahoma History" or "Gazi University Archive System" or "GB3D Type Fossils" or "GBRMPA eLibrary" or "GEO-LEOe-docs" or "Geograph British Isles" or "Georgetown Law Scholarly Commons" or "German Medical Science" or "Getty Search Gateway" or "GFZ German Research Centre for Geosciences" or "Ghent University Academic Bibliography" or "Giessener Elektronische Bibliothek" or "Glasgow Theses Service" or "Global Performing Arts Database" or "Goddard Library Repository" or "Goldsmiths Research Online" or "Golestan University of Medical Sciences Repository" or "Gothic Past" or "Graduate School Section of the eTD database" or "Graph Drawing E-print Archive" or "Greater Cincinnati Memory Project" or "GREDOS" or "Greenwich Academic Literature Archive" or "Greifswalder Digitales Informationssystem" or "Grey Literature Digital Library at the National and Kapodistrian University of Athens" or "Griffith Open"

SC
Supplemental content to
J Med Libr Assoc. Apr;108(2):dx.doi.org/10.5195/jmla.2020.856
www.jmla.mlanet.org
© Demetres, Delgado, Wright 2020

or "Griffith Research Online" or "GSI Repository" or "Guaiaca" or "Gunma University Academic Information Repository" or "Gutenberg Open" or "Gutenberg Qualify" or "HAL - Audencia Group" or "HAL - Lille 3" or "HAL AMU" or "HAL Descartes" or "HAL Evry" or "HAL Mines Nantes" or "HAL-Artois" or "HAL-CEA" or "HAL-CIRAD" or "Hal-Diderot" or "HAL-Ecole des Ponts ParisTech" or "HAL-EMSE" or "HAL-ENS-LYON" or "HAL-HCL" or "HAL-IFPEN : Archive ouverte IFP Energies nouvelles" or "HAL-IN2P3" or "HAL-INERIS" or "HAL-INRAP" or "HAL-INSA Toulouse" or "HAL-Inserm" or "HAL-INSU" or "HAL-IRD" or "HAL-Lyon 3" or "HAL-MINES ParisTech" or "HAL-OBSPM" or "HAL-Paris 13" or "HAL-Paris1" or "HAL-Pasteur" or "HAL-Polytechnique" or "HAL-Rennes 1" or "HAL-SUPELEC" or "HAL-uB" or "HAL-UJM" or "HAL-UNICE" or "HAL-UNILIM" or "HAL-Univ-Nantes" or "Bretagne Occidentale" or "HAL-UPMC" or "HamaMed-Repository" or "HANYANG Repository" or "Hanze UAS repository" or "Harare Institute of Technology Repository" or "Harvard College Thesis Repository").mp.

12 ("Harvard Dataverse" or "Harvard Smithsonian Digital Video Library" or "HBO Knowledgebase" or "Health Sciences Research Commons" or "Hebrew Books" or "Hector Kobbekaduwa Agrarian Research and Training Institute Repository" or "Hedatuz" or "HEFT Repository" or "Heidelberger Dokumentenserver" or "HELIOS Repository" or "Hellenic National Archive of Doctoral Dissertations" or "Helmholtz Zentrum" or "Helsebibliotekets Research Archive" or "Helsingin yliopiston digitaalinen arkisto" or "Heritage Repository" or "HERMES-IR" or "Heythrop College Publications" or "Brage" or "Hiroshima Associated Repository Portal" or "Hiroshima Shudo University Institutional Ripository System" or "Historic American Sheet Music" or "HKU Scholars Hub" or "HM Digital" or "Hochschulschriftenserver" or "Hodges University Institutional Repository" or "Hokkaido University Collection of Scholarly and Academic Papers" or "Hokkaido University of Education Repository" or "Hope's Institutional Research Archive" or "Horizon / Pleins textes" or "HSF Brage" or "HSH Brage publication archive" or "HSN Open archive" or "Humboldt Digital Scholar" or "Hungarian Electronic Library" or "Hungarian Veterinary Archive" or "Huskie Commons" or "Hyokyo Educational and Academic Resources for Teachers" or "Hyper Article en Ligne" or "HZB Repository" or "HZG Publication Database" or "HZSK Repository" or "I-Revues" or "IAIN Sunan Ampel Repository" or "IAU Scholarly Archive Database" or "IBB PAS Repository" or "Ibiblio" or "IBSS Repository" or "IC-online" or "ICM - DIR - Zasoby Polskie" or "ICOMOS Open Archive" or "ICPBS Digital Collections" or "ICTP Open Access Archive" or "Idaho State Historical Society Digital Collections" or "IDEP Document Server" or "IDRC Digital Library" or "IDS OpenDocs" or "IFAPA - Servifapa" or "IFE Brage" or "IFPRI Knowledge Repository" or "Igdir University" or "IIASA PURE" or "IIT Roorkee Repository" or "ILC4CLARIN repository of language resources and tools" or "Illinois Air Photo Imagebase" or "Illinois Digital Archives" or "Illinois Digital Environment for Access to Learning and Scholarship Repository" or "Illinois Electronic Documents Initiative" or "Illinois Harvest" or "IMechE Virtual Archive" or "Imperial War Museum Collections and Research" or "IMT E-Theses" or "INBA Digital" or "Indian Academy of Sciences: Publications of Fellows" or "Indian Institute of Astrophysics Repository" or "Indian Institute of Management Kozhikode Digital Library" or "Indiana Historical Society Digital Image Collections" or "Indonesian Institute of the Art Yogyakarta" or "Influenza Training Digital Library" or "Informatics@Edinburgh - Reports Series" or "INFORUM Conference Proceedings" or "Infoscience" or "Infoteca-e" or "INRIA a CCSD electronic archive server" or "INSIDE Idaho" or "Institut National Polytechnique de Toulouse (Theses)" or "Institute for Christian Studies" or "Institute for Social Research in Zagreb (ISRZ) Repository" or "Institute of Transport Research:Publications" or "Institute of Volcanology and Seismology FEB RAS Repository" or "Institute Repository of TianJin Institute of Industrial Biotechnology" or "Institutional Knowledge at Singapore Management University" or "InsubriaSPACE" or "INtellectual property in DIGital form available online in an Open environment" or "Intellectual Repository of Rajamangala University of

Supplemental content to
J Med Libr Assoc. Apr;108(2):dx.doi.org/10.5195/jmla.2020.856
www.jmla.mlanet.org
© Demetres, Delgado, Wright 2020

Technology Lanna" or "Intellectual Repository Production UCLA" or "Intellectum" or "Inter-American Development Bank Repository" or "InterNano Nanomanufacturing Repository" or "International Migration, Integration and Social Cohesion online publications" or "International University of Africa Repository").mp.

13 ("INTRAC Resource Centre" or "Invenia Repository for Technological Innovation" or "Inventaire du patrimoine" or "IO PWr" or "Iowa Publications Online" or "Iowa Research Online" or "IPACS Electronic library" or "IPVC Repository" or "IR of Centre for Egyptological Studies of Russian Academy of Sciences" or "IR South Eastern University of Sri Lanka" or "IR@CECRI" or "IR@Goa University" or "IR@LSU" or "IR@NPL" or "IRI repository" or "IRIS - Institutional Research Information System of the University of Trento" or "IRIS Catalogo dei prodotti della ricerca scientifica LUISS" or "Irish Health Repository" or "ISFOL OA" or "Isik University Academic Open Access" or "Island Archives.ca at the University of Prince Edward Island" or "IslandScholar" or "ISM Science Box" or "Ispektra Digital Collection" or "ISS Library" or "IssueLab" or "IST PubRep" or "Istanbul Arel University Digital Archive" or "Istanbul Bilgi University Library Open Access" or "Istanbul Bilim University" or "ISU Electrical and Computer Engineering Archives" or "ISU ReD: Research and eData" or "IUBio Archive" or "IUBio Software Archive" or "IUPUIScholarWorks" or "IUScholarWorks" or "IWMI Publications" or "J. Willard Marriott Library Digital Collections" or "Jable. Archivo de prensa de Canarias" or "Jagiellonian Digital Library" or "JAIST Repository" or "JAMSTEC Repository" or "Japan International Cooperation Agency Research Institute Repository" or "Japanese Institutional Repositories Online(JAIRO)" or "JCF Seek" or "Jean Monnet Working Papers" or "JIKEI Repository" or "Jisc Repository" or "JKU ePub" or "Jobim" or "Joetsu University of Education Repository" or "Johns Hopkins Bloomberg School of Public Health's OpenCourseWare" or "Johnson and Wales University Scholars Archive" or "Johnson Technical Reports Server" or "Jorum Open" or "JPL Technical Reports Server" or "JRC Publications Repository" or "JSC Digital Image Collection" or "JSC Reduced Gravity Program Photographs" or "JScholarship" or "Juelich Shared Electronic Resources" or "Jukuri" or "Julkari" or "K-Developedia(KDI School) Repository" or "K-State Research Exchange" or "Kaiserslauterer uniweiter elektronischer Dokumentenserver" or "KAIST Open Access Self-Archiving System" or "Kaleidoscope Open Archive" or "Kansas State Publications Archival Collection" or "Kanto Gakuin University IR" or "KARI e-repository" or "KarUSpace" or "Kashan University of Medical Sciences Repository" or "Katholieke Hogeschool Kempen Theses" or "Kauno Kolegija Repository" or "KAUST Digital Archive" or "KCA Academic Commons" or "KCE Repository" or "KDI School Archives" or "KDK Repository" or "KeiO Academic Resource Archive" or "KhartoumSpace" or "KHIODA" or "KIM Repository" or "Kinematic MOdels for Design Digital Library" or "King's Research Portal" or "Kinki University Academic Resource Repository" or "KINU Repository" or "Kirchlicher Dokumentenserver" or "Kirchlicher DokumentenServer der AKThB und des VkwB" or "Kitami Institute of Technology Repository" or "KITopen" or "KNAW Repository" or "Knowledge Repository of HFCAS" or "Knowledge Repository of Indian Institute of Horticultural Research" or "Knowledge Repository of Institute Of IHEP,CAS(IHEP OpenIR)" or "Knowledge Repository of National Science Library, CAS" or "Knowledge Repository of SEMI,CAS" or "Knowledge Repository Open Network" or "Knowledge, Imaginary, Discovery, Sharing").mp.

14 ("KnowledgeBank at OSU" or "knustspace" or "Kochi Rehabilitation Institute Repository(POST)" or "Kochi University of Technology Academic Resource Repository" or "Kokushikan Knowledge Integrated Service System" or "Kolbuszowa Digital Library" or "Konrad Zuse Internet Archive" or "Konstanzer Online-Publikations-System" or "Korea Institute of International Economic Policy Repository" or "Korea Thermophysical Properties Data Bank" or "KoreaMed Synapse" or "Kosmopolis" or "KOTRA" or "KovsieScholar" or "KRIBB Repository" or "Krigsskolens publiseringsarkiv" or "KrishiKosh" or "KRIVET Repository" or "Krosnienska Biblioteka Cyfrowa

Supplemental content to
J Med Libr Assoc. Apr;108(2):dx.doi.org/10.5195/jmla.2020.856
www.jmla.mlanet.org
© Demetres, Delgado, Wright 2020

(Krosno Digital Library)" or "KTUePubl (Repository of Kaunas University of Technology)" or "KU ScholarWorks" or "Kujawsko-Pomorska Biblioteka Cyfrowa (Kujawsko-Pomorska Digital Library)" or "KUMeL Repository" or "Kun Shan University Digital Resource Repository" or "kydl OAI Archive" or "Kyoto University Research Information Repository" or "Kyoto Women's University Academic Information Repository" or "KYPSELI" or "Kyushu University of Health and Welfare Repository" or "La Trobe University Research Online" or "Lake Forest College Publications" or "Lake Victoria Basin Commission (LVBC) Repository" or "Lakehead University Knowledge Commons" or "Language Box" or "Laospace" or "Latin American Open Archives Portal" or "Lauda" or "LBS Research Online" or "Learning Exchange" or "Leeds Trinity University" or "Lehigh Preserve" or "Leicester Research Archive" or "LEKYTHOS" or "Leodis - A photographic archive of Leeds" or "Les theses electroniques" or "Letter from America by Alistair Cooke" or "Levadia Central Public Library Repository" or "Liberal Arts Scholarly Repository (LASR)" or "Librarians' Digital Library" or "Library and Archives Canada" or "Library and Historical Collections Digital Resources" or "Libre Acces aux Rapports Scientifiques et Techniques" or "Liburuklik Biblioteca Digital Vasca" or "LIFE+ MarPro Repository" or "Lincoln University Research Archive" or "LINDAT-CLARIN repository" or "LingBuzz" or "Linguistic Electronic Archive" or "Lirias" or "Livre Saber - Repositorio Digital de Materiais Didaticos" or "Livro Aberto" or "LJMU Research Online" or "Lodz University of Technology Repository" or "London Met Repository" or "LOUISiana Digital Library Server Respository" or "Lowcountry Digital Library" or "Loyola eCommons" or "LSBU Research Open" or "LSE Research Online" or "LSE Theses Online" or "LSHTM Data Compass" or "LSHTM Research Online" or "LSTM Online Archive" or "Lublin University of Technology Digital Library" or "Lucerne Open Repository" or "Ludovika Digital Knowledge Repository and Archive (LUDITA)" or "LUISSearch" or "Lumbung Pustaka UNY (UNY Repository)" or "Lunar and Planetary Institute, Resources" or "Lund University Publications" or "LUTPub" or "Macquarie University ResearchOnline" or "MacSphere" or "Madan Puraskar Pustakalaya" or "MAGiC ARC Repository" or "Mahatma Gandhi University Theses Online" or "MAnnheim DOCument Server" or "Manuscripts and Archives Digital Image Database" or "Mario Mgulunde Learning Resource Centre Repository" or "Marmara University Open Archive Repository" or "Marshall Digital Scholar" or "Marshall Foundation Digital Library" or "Marshall Technical Reports Server" or "Mason Archival Repository Service" or "Massey Research Online" or "MatDB" or "Material Properties Open Database" or "Mathematics in Medicine Study Groups" or "Max Planck Institute for the Study of Societies Publications" or "Max Planck Society eDoc Server").mp.

15 ("Mazowiecka Biblioteka Cyfrowa" or "MB IPB Repository" or "MCStor" or "MediaTUM" or "MedUni Wien ePub" or "Meiji Repository" or "Memoria digital de Canarias" or "Memorial University Newfoundland Digital Archive Initiative" or "Mendeley Data" or "Mertz Digital Collections @ New York Botanical Garden Digital Library" or "Metabiblioteca-Biblioteca Digital Libros Abiertos" or "Metabolights" or "Metadata on Internet Documents" or "Metropolitan Travel Survey Archive" or "MF Brage" or "Michael Servetus Research" or "Microsoft Research Catalog" or "Mie University Scholarly E-collections" or "Minds@University of Wisconsin" or "Mineralis" or "Minority Health Archive" or "Mississippi State University Libraries ETD database" or "Mittuniversitetets Publikationer" or "Miyagi University of Education Repository" or "MKU Rwanda Repository" or "Modern Languages Publications Archive" or "MODIYA Project" or "Mona Online Research Database" or "Montagne@Doc" or "Morska Biblioteka Cyfrowa (Maritime Digital LIbrary)" or "MOspace" or "MOST Digital Library" or "Mount Saint Vincent University" or "Mountain Forum" or "MPG.PuRe" or "MSpace at the University of Manitoba" or "Multimedia ONline ARchiv CHemnitz" or "Munich RePEc Personal Archive" or "Munin - Open Research Archive" or "Muroran-IT Academic Resource Archive" or "Museo Virtual del Seguro" or "Mutopia" or "MyManuskrip: Digital Library of Malay Manuscripts (Pustaka Digital Manskrip Melayu)" or "Mzumbe University Scholar Repository" or

Supplemental content to
J Med Libr Assoc. Apr;108(2):dx.doi.org/10.5195/jmla.2020.856
www.jmla.mlanet.org
© Demetres, Delgado, Wright 2020

"Nagasaki university's Academic Output SITE" or "Nagoya City University Repositories" or "Nagoya Institute of Technology Repository System" or "Nagoya Repository" or "Najran University's Repository" or "Nano Archive" or "Nara National Research Institute for Cultural Properties Repository" or "Nara Womens University Digital Information Repository" or "NASA Dryden Technical Reports Server" or "NASA Technical Reports Server" or "Nasi u FP" or "National Academies Press" or "National Central University Library Electronic Thesis & Dissertation System" or "National Digital Library Polona" or "National Documentation Centre on Drug Use" or "National Engineering Education Delivery System" or "National Institute for Fusion Science (NIFS-Repository)" or "National Institute of Fitness and Sports in Kanoya Repository" or "National Jukebox" or "National Library of Australia Digital Object Repository" or "National Library of Serbia - Digital Object Identifier Repository" or "National Museum of Ethnology Repository" or "National Museum of Japanese History Repository" or "National Repository of Dissertations in Serbia" or "National Repository of Grey Literature" or "National Repository of Open Educational Educational Resources" or "National Research Database of Zimbabwe" or "National Science Digital Library" or "National Taipei University of Education Repository" or "National Taipei University of Nursing and Health Sciences Repository" or "National University of Kashsiung Repository" or "Natural History Museum Repository" or "NCSU Technical Reports Repository" or "Neliti" or "NELLCO Legal Scholarship Repository" or "Nemertes" or "NERC Open Research Archive" or "New Bulgarian University Scholar Electronic Repository" or "New College of Florida Digital Collections" or "New York University Faculty Digital Archive" or "Newcastle University E-Prints" or "NIFU Open Access Archive" or "Niigata College of Nursing Repository" or "Niigata Regional Repository" or "NILU Brage" or "NIOZ Repository" or "NISCAIR Online Periodical Repository" or "NIST Repositories" or "NIT, Tsuyama College-Repository" or "NITTAIDAI Repository" or "NLR Reports Repository" or "NM-AIST Repository" or "NMH - Brage" or "NOFIMA repostitory" or "Nordic Africa Institute" or "Northeast Massachusetts Digital Library: Imagining History Collections").mp.

16 ("Northumbria Research Link" or "Norwegian Geotechnical Institute (NGI) Digital Archive" or "Norwegian Institute of Public Health Open Repository" or "Nottingham eTheses" or "Nottingham Research Data Management Repository" or "Nowohucka Biblioteka Cyfrowa (Nowa Huta Digital Library)" or "NPUE IR" or "NRC Publications Archive" or "NSTDA Knowledge Repository" or "NSU Works" or "NTNU Open" or "NUPI Research Online" or "NuSpace" or "NuStone" or "NYPL Map Warper" or "Oak Repository-KIST" or "OAPEN Library" or "OAR@UM" or "Occidental College Scholar" or "OceanDocs" or "OceanRep" or "OCLC Research Publications" or "Odessa State Academy of Civil Engineering and Architecture electronic Repository" or "Odum Institute Data Archive" or "Office of Scientific & Technical Information" or "Ohio Digital Resource Commons - Marietta College" or "OhioLINK Digital Resource Commons" or "OhioLINK Electronic Theses and Dissertations" or "OhioLINK Electronic Thesis and Dissertation Center" or "OhmDok" or "OIE Repository" or "Okayama Prefectural University ePublications Repository" or "Okayama University Scientific Achievement Repository" or "Okinawa Repository Integrated Open-Access Network" or "Oldenburger Online Publikations Server" or "On-Line University Writings - Universit√É¬§t Halle-Wittenberg" or "Online Archive of University of Virginia Scholarship" or "Online Publikationen der Universit√É¬§t Stuttgart" or "Online Publikationsserver OPUS der Hochschule Osnabr√É¬ºck" or "Online Repository of Birkbeck Institutional Theses" or "Online Research @ Cardiff" or "Online Research Database In Technology" or "Online-Publikationssystem der Bauhaus-Universit√É¬§t Weimar" or "ONMUIR" or "Open Access @ Bingol University" or "Open Access Collection of International and Scholarly Papers" or "Open Access Library (Repository)" or "Open Access LMU" or "Open Access to Odia Books" or "Open Archief van VIOE-publicaties" or "Open Archive for Conferences held by the Department of Mathematics - Politecnico di Milano" or "Open Archive of

© Demetres, Delgado, Wright 2020

Northern State Medical University (Arkhangelsk)" or "Open Archive Toulouse Archive Ouverte" or "Open Archive University of Naples L'Orientale" or "Open Digital Archive at Oslo and Akershus University College" or "Open FHR Archive" or "Open Knowledge Environment of the Caribbean" or "Open Knowledge Repository" or "Open Library Archives of Kagawa University" or "Open Marine Archive" or "Open Repositories 2008 Publications" or "Open Repository and Bibliography - Luxembourg" or "Open Repository of Keldysh Institute of Applied Mathematics of RAS" or "Open Research Exeter" or "Open Research Online" or "Open Resources" or "Open Thesis" or "Open Video Project" or "Open-access Collection and Electronic Archives for academic Navigator" or "Open-Access-Repositorium der TH Wildau" or "OpenAccess@IKU" or "OpenArchive@CBS" or "OpenArchive@GSOM " or "OpenBU" or "OpenDEPOT.org" or "OpenEmory" or "OpenFields" or "OpenGrey Repository" or "OpenKnowledge@NAU" or "OpenMED@NIC" or "OpenPub" or "OpenSALDRU" or "OpenSIUC" or "OpenSky" or "OpenstarTs" or "OpenUCT" or "OpenUniud" or "Opin visindi" or "OPLex" or "oPUB" or "Opus: Research and Creativity at IPFW" or "ORE Digital Library" or "Osaka Prefecture University Education and Research Archives" or "Osaka University Knowledge Archive" or "Oslo University College's PROJECT.iu.hio.no" or "Osmania University Digital Library [OUDL]" or "OstDok - Osteuropa-Dokumente online" or "Otago University Research Archive" or "Otwarte Repozytorium Nauk Historycznych LECTORIUM" or "Ounongo Repository").mp.

17 ("Our Homes are Bleeding (Nos foyers saignent)" or "oURspace" or "Oxfam Policy & Practice" or "Oxford Text Archive" or "Oxford University Research Archive" or "OZone provided by Ontario Scholars Portal" or "P-arch" or "Pacific Archive of Digital Data for Learning and Education" or "Padua@research" or "Padua@thesis" or "Pandektis" or "PANDEMOS" or "Papers Past" or "Pasundan Repository" or "PDBj (Protein Data Bank Japan)" or "PDXScholar" or "PEAK Digital" or "Pedagogical Digital Library" or "PeFprints" or "People @ FH Burgenland" or "PEPITE Panorama des productions universitaires" or "PePSIC - Electronic Psychology Journals" or "Pergamos Digital Library" or "Permanent Hosting, Archiving and Indexing of Digital Resources and Assets" or "Perseus Digital Library" or "PhilPapers" or "PhilSci Archive" or "PiezoMat.org" or "PIK Publications" or "Plymouth Electronic Archive and Research Library" or "Plymouth Marine Science Electronic Archive (PlyMEA)" or "Podkarpacka Digital Library" or "Podlaska Digital Library" or "POLNEP Repository" or "PolyPublie" or "Pomeranian Digital Library" or "Portal de Tesis Electronicas Chilenas" or "Portal de Tesis Latinoamericanas" or "Portal do Conhecimento" or "Portal Garuda STMIK IBBI (STMIK IBBI Repository)" or "Portal to Texas History" or "Portale Vico" or "Portsmouth Research Portal" or "Postgrado de la FF. CC. AA. Universidad de Guayaquil" or "Predicted Crystallography Open Database" or "Prefectural University of Hiroshima Repository" or "Presidencia de la Republica. Secretaria de Derechos Humanos para el Pasado Reciente - Coleccion digital Prensa" or "ProdInra" or "Projeto Maxwell" or "Prometheus-Academic Collections" or "Propylaeum-DOK" or "PSU Knowledge Bank" or "PTSL UKM Repository" or "pub H-BRS - Publikationsserver der Hochschule Bonn-Rhein-Sieg" or "Pubblicazioni Aperte Digitali Interateneo Sapienza" or "Public Central Library of Serres Repository" or "Public Digital Archive of Agnieszka Osiecka" or "Publication Database of the Vienna University of Technology" or "PUblication MAnagement" or "Publication Server of the Aachen University of Applied Sciences" or "Publications at Bielefeld University" or "Publications Et Travaux Acad√É¬©miques de LorrainE" or "Publications from Karolinska Institutet" or "Publications of the Interactive and Cooperative Technologies Lab" or "Publications Open Repository TOrino" or "Publications Repository" or "PubliCatt" or "PubLIS Cologne - Repository of the Institute of Information Science Cologne University of Applied Science" or "Publishing Network for Geoscientific and Environmental Data" or "Purdue E-Scholar" or "Purdue University Libraries E-archives" or "Pusan National University Hospital Repository" or "Qazvin University of Medical Sciences

JMLA Journal of the Medical Library Association

Supplemental content to
J Med Libr Assoc. Apr;108(2):dx.doi.org/10.5195/jmla.2020.856
www.jmla.mlanet.org
© Demetres, Delgado, Wright 2020

Repository" or "QSpace at Queen's University" or "Qucosa" or "Queen Margaret University eResearch" or "Queen Mary Research Online" or "Queen's Papers on Europeanisation" or "Queen's University Research Portal" or "Queensland Department of Agriculture and Fisheries eResearch Archive" or "R-libre" or "R-Space, Korea Rural Economic Institute" or "R4D" or "Radboud Repository" or "Radom Digital Library" or "RAIITH" or "Rajamangala University of Technology Phra Nakhon Intellectual Repository" or "Rare Books and Special Collections Digital Library" or "RaY - Research at York St John" or "RCTI" or "Real Academia Nacional de Medicina: Biblioteca Digital" or "Recherche uO Research" or "Red de Bibliotecas Virtuales de Ciencias Sociales de Am√É¬©rica Latina y El Caribe" or "Red Island Repository" or "Red Mexicana de Repositorios Institucionales" or "Redalyc" or "ReDIUC" or "RediUMH" or "Repositoorium E-ait" or "Repositori d'Objectes Digitals per a l'Ensenyament la Recerca i la Cultura").mp.

18 ("Repositori Institucional de la Universitat Jaume I" or "Repositori Institucional URV" or "Repositori Institusi Kementerian Pendidikan dan Kebudayaan" or "Repositori Obert de Coneixement de l'Ajuntament de Barcelona" or "Repositori Obert UdL" or "Repositori Universitas Bhayangkara Jakarta Raya" or "Repositorio Academico Digital" or "Repositorio CESA" or "Repositorio CUDI" or "Repositorio da Universidade de Lisboa" or "Repositorio de Acceso Abierto EDUMED" or "Repositorio de Ciencias Agropecuarias y Ambientales del Noroeste Argentino" or "Repositorio de Digital Institucional - UCSG" or "Repositorio de la UNED" or "Repositorio de la UNIA" or "Repositorio de la Universidad de Cuenca" or "Repositorio de la Universidad de Puerto Rico" or "Repositorio de la Universidad Estatal a Distancia de Costa Rica" or "Repositorio de Material Educativo" or "Repositorio de Tesis USAT" or "Repositorio Digital" or "Repositorio Documental de la Universidad de Valladolid" or "Repositorio Documental de la Universidad Nacional Francisco" or "Repositorio Documental UMNG" or "Repositorio Educativo Digital Universidad de Occidente" or "Repositorio EMI+D" or "Repositorio Hipermedial de la Universidad Nacional de Rosario" or "Repositorio Insitucional de la Universidad Nacional de Salta" or "Repositorio Insitucional del Ministerio" or "Repositorio INSP - Banco de Tesis" or "Repositorio Institucional" or "Repositorio OAI Biblioteca Digital Universidad Nacional de Cuyo" or "Repositorio UASB-DIGITAL" or "Repositorio UC" or "Repositorio UCAL" or "Repositorio Universidad de Belgrano.Argentina" or "Repositorio Universidad Sergio Arboleda" or "Repositorio Universitario de la DGTIC" or "Repositorio USB" or "Repositorium und Bibliografie der Hochschule Reutlingen" or "Repository at St. Cloud State" or "Repository Belarusian State Medical University" or "Repository CWI Amsterdam" or "Repository Faculty of Agriculture University of Zagreb" or "Repository of Agricultural Research Outputs" or "Repository of Almaty Management University" or "Repository of Baranovichi State University" or "Repository of Belarusian National Technical University (BNTU)" or "Repository of Belarusian State University of Culture and Arts" or "Repository of Centre for Open Science" or "Repository of Department of Physics in Osijek" or "Repository of Dnipropetrovsk Medical Academy" or "Repository of Economics faculty in Split" or "Repository of Educational Research and Practice in Niigata" or "Repository of Faculty of Chemical Engineering and Technology University of Zagreb" or "Repository of Faculty of Civil Engineering Osijek" or "Repository of Faculty of Geotechnical Engineering" or "Repository of Faculty of Humanities and Social sciences, University of Split" or "Repository of Faculty of Kinesiology, University of Zagreb - KIFoREP" or "Repository of Faculty of Metallurgy University of Zagreb" or "Repository of Faculty of Pharmacy and Biochemistry University of Zgreb" or "Repository of Faculty of Science, University of Zagreb" or "Repository of FERIT Osijek" or "Repository of Gomel State University" or "Repository of Grodno State Medical University" or "Repository of Kazimierz Wielki University" or "Repository of L.N. Gumilyov Eurasian National University" or "Repository of Mozyr State Pedagogical University named after I.P. Shamyakin" or "Repository of Nicolaus Copernicus University" or "Repository of NNCT (Nagano National College

Supplemental content to
J Med Libr Assoc. Apr;108(2):dx.doi.org/10.5195/jmla.2020.856
www.jmla.mlanet.org
© Demetres, Delgado, Wright 2020

of Technology)" or "Repository of Open Access Documents" or "Repository of Open access Scholarly E-collections" or "Repository of Polessky State University " or "Repository of Polytechnic in Pozega" or "Repository of Polytechnic Nikola Tesla" or "Repository of Rzeszow University" or "Repository of the Academy of Arts, University of Osijek" or "Repository of the Academy's Library" or "Repository of the Catholic Faculty of Theology University of Zagreb" or "Repository of the College in Slavonski Brod" or "Repository of the Czech Academy of Sciences" or "Repository of the Department of Chemistry, Osijek" or "Repository of the Faculty of Chemistry and Technology, University of Split" or "Repository of the Faculty of Civil Engineering, Architecture and Geodesy, University of Split" or "Repository of the Faculty of Education" or "Repository of the Faculty of Food Technology and Biotechnology" or "Repository of the Faculty of Humanities and Social Sciences Osijek" or "Repository of the Faculty of Transport and Traffic Sciences" or "Repository of the Gomel State Medical University" or "Repository of the RRiF College of Financial Management" or "Repository of the School of Dental Medicine University of Zagreb" or "Repository of the Sestre milosrdnice University Hospital Center" or "Repository of the University of Dubrovnik" or "Repository of the University of Ljubljana" or "Repository of the University of Nagasaki" or "Repository of the University of Rijeka" or "Repository of the University of Rijeka, Department of Biotechnology" or "Repository of the University of Rijeka, Faculty of Economics" or "Repository of the University of Rijeka, Faculty of Humanities and Social Sciences" or "Repository of the Vitebsk State University named after P.M.Masherov").mp.

19 ("Repository of the Yaroslav Mudryi National Law University" or "Repository of University of Nova Gorica" or "Repository of University of Primorska" or "Repository of University of Zagreb, Centre for Croatian Studies" or "Repository of Vinnytsia National Technical University" or "Repository of Vinnytsya National Agrarian University" or "Repository of Zagreb School of Business" or "Repository Open Access to Scientific Information from Embrapa" or "Repository Poltekkes Kemenkes Yogyakarta" or "Repository ST3 Telkom" or "Repository TU/e" or "Repository UIN Sumatera Utara" or "Repository UMMI" or "Repository Universitas " or "Repository@Napier" or "Repository@USM" or "reposiTUm" or "Repozitory of E.A.Buketov Karaganda State University" or "Repozytorium Cyfrowe UTP w Bydgoszczy" or "Repozytorium Eny Politechnika" or "Repozytorium Instytucjonalne Krakowskiej Akademii" or "Repozytorium PJWSTK / PJIIT Repository" or "Repozytorium Politechniki Krakowskiej" or "University of Bialystok Repository" or "University of Warsaw Repository" or "Knowledge Repository of the Wroclaw University of Technology" or "RERO DOC Digital Library" or "Research Archive and Digital Asset Repository" or "Research Archive of Indian Institute of Technology Hyderabad" or "Research Art Design Architecture Repository" or "Research at Sofia University" or "Research at the University of Wales, Newport" or "Research Commons@Waikato" or "Research Exchange" or "Research in Architecture, Design and Conservation" or "Research Institute for Humanity and Nature Repository" or "Research Online @ ECU" or "Research Online at MacEwan" or "Research Papers in Economics" or "Research Showcase @ CMU" or "Research Support Scheme - Central European University" or "Research+rmuts" or "ResearchArchive at Victoria University of Wellington" or "ResearchOnline@Avondale" or "ResearchOnline@GCU" or "ResearchOnline@JCU" or "ResearchOnline@ND" or "ResearchSpace@Auckland" or "ResearchSpace@UKZN" or "Resena Historica del Teatro en Mexico 2.0-2,1 Sistema de la critica teatral" or "Resource Repository" or "ReStore repository" or "Reti Medievali Open Archive" or "Riberdis" or "Rice Digital Scholarship Archive" or "Riksantikvarens vitenarkiv" or "RIT Digital Media Library" or "RIT Scholar Works" or "RiuNet" or "RIUVic" or "ROAR at University of East London" or "Rolnicza Biblioteka Cyfrowa (Agricultural Digital Library)" or "Roskilde Universitetscenter's Digitale Arkiv" or "Royal Holloway Data Archive" or "Royal Holloway Research" or "Rpion College Senior Showcase" or "RRUFF Project" or "RSpace" or "Rubber Research Institute Repository" or "RUIdeRA"

or "RUKSOR(Repository of University of Kitakyushu Stacked by Original Resources)" or "Rural Access Library" or "Rutgers University Community Repository" or "RVC Research Online" or "S@L: Scholarship at Lesley" or "SaarDok-Datenbank" or "Sabanci University Research Database" or "Saber UCAB" or "Sabzevar University of Medical Sciences Electronic Publications" or "Saitama Institute of Technology Academic Collections" or "Saitama University Cyber Repository of Academic Resources" or "SAM : Science Arts" or "Sandomierz Diocese Digital Library" or "Sandra Day O'Connor College of Law Faculty Scholarship Repository" or "Sanok Digital Library" or "Sapientia" or "sapporo medical university Information and KnOwledge Repository)" or "SAS-SPACE" or "SAUL Archive" or "Savoirs UdeS" or "Scholar Commons" or "ScholarBank@NUS" or "Scholarly Commons" or "Scholarly Materials And Research @ Georgia Tech" or "ScholarlyCommons@Penn" or "Scholars Commons @ Laurier" or "Scholars' Mine" or "ScholarsArchive@OSU" or "Scholarship @ Cornell Law" or "Scholarship at Parkland").mp.

20 ("Scholarship at UWindsor" or "Scholarship Repository of Florida Institute of Technology" or "Scholarship@Claremont" or "Scholarship@Western" or "ScholarSpace at University of Hawai'i at Manoa" or "ScholarSphere" or "ScholarWorks" or "School of Business IPB Repository" or "School of Information, University of Texas at Austin" or "Science and Religion Dialogue Prints" or "Science Attic" or "Science Media" or "Scientific documents from the Saarland University," or "Scientific Open-access Literature Archive and Repository" or "Scientific Publications of the University of Toulouse II Le Mirail" or "Scientific Repository" or "Scioteca" or "Scivee TV" or "SciVie" or "SCOAP3 Repository" or "Scriptorium" or "SCU Digital Collections" or "Search4Dev" or "Seigakuin Repository for Academic Archive" or "SelectedWorks @ Oklahoma City University School of Law" or "Seminole State College of Florida Digital Collections" or "Semmelweis Repository" or "Senshu University Institutional Reposigory " or "Seoul Metropolitan Library" or "Serbian Academy of Science and Arts Digital Archive" or "SERPENT Image & Video Database" or "Servicios Bibliotecarios de la Universidad de Los Andes" or "SFA ScholarWorks" or "ShareGeo Open" or "SHAREOK repository" or "Sheffield Hallam University Research Archive" or "Sheffield Hallam University Research Data Archive" or "Shiga University of Medical Science Repository BIWAKO" or "Shimane University Web Archives of kNowledge" or "ShodhGanga: A reservoir of Indian theses" or "Shujitsu Digital Information Repository" or "Silesian University of Technology Digital Library" or "SINTEF Open" or "SIOExplorer Digital Library Project" or "SISSA Digital Library" or "Sistem Informasi Tugas Akhir" or "SJSU ScholarWorks" or "Skemman" or "SNHU Academic Archive" or "SNU Health Repository" or "SNU Open Repository and Archive" or "SOAS Research Online" or "Social Science Cyber Library" or "Social Science Research Network" or "SOE Repository of Dissertations" or "SOE Repository of Publications" or "Solent Electronic Archive" or "SOURCE: Sheridan Scholarly Output Undergraduate Research Creative Excellence" or "South Africa Data Archive" or "South Carolina State Documents Depository" or "SRUC Repository" or "SSPAL.doc" or "St George's Online Research Archive" or "St Mary's University Open Research Archive" or "St. Luke's College of Nursing Repository" or "Stain Salatiga Online Repository" or "Stark Center E-Archive" or "State Library of Massachusetts" or "Statistics Online Computational Resources" or "Stellenbosch University SUNScholar Repository" or "Stirling Online Repository for Research Data" or "STMIK GI MDP" or "STORE - Staffordshire Online Repository" or "Student Scholar Archive" or "SU-Portal" or "Sudan Open Archive" or "SUMMA. Repositorio Documental UPSA" or "SUNDigital Collections" or "Suquia" or "Suranaree University of Technology Intellectual Repository" or "Surrey Research Insight" or "Surugadai University Academic Information Repository" or "Sussex Research Online" or "SUZA REPOSITORY" or "Swedish Institute of Computer Science Publications Database" or "Swedish School of Sport and Health Sciences" or "Swinburne Image Bank" or "Swinburne Research Bank" or "SWPS's and WSNHiD's Repository (Repozytorium SWPS i WSNHiD)" or "Sycamore Scholars" or "Sydney eScholarship" or "Syracuse

Supplemental content to
J Med Libr Assoc. Apr;108(2):dx.doi.org/10.5195/jmla.2020.856
www.jmla.mlanet.org
© Demetres, Delgado, Wright 2020

University Research Facility and Collaborative Environment" or "SysSec Project Publications" or "System Competence Area Document Server" or "SZTAKI Publication Repository" or "Taiwan Agricultural History Digital Archives During the Japanese Colonial Period" or "Tama-Kura: Tama University" or "Tanzania Climate Change Information Repository" or "Tarnobrzeska Biblioteka Cyfrowa" or "Tarnow Digital Library").mp.

21 ("Tavistock and Portman Staff Publications Online" or "Tea Research Institute Repository" or "Teaching & Learning Research Programme Publications" or "Technical Registry" or "TECNALIA Publications" or "TED Ankara College IB Thesis" or "Temple University Electronic Dissertations" or "Temple University Libraries: Digital Colletions" or "Tennessee Historical and Regional Collections" or "Tennessee Research and Creative Exchange" or "Tesis Doctorals en Xarxa" or "Tesis Electronica UACh" or "Tesis Electronicas de la Universidad de Chile" or "Texas Digital Library Repository" or "Texas ScholarWorks" or "The Academy of Public Administration under the aegis of the President of the Republic of Belarus" or "The Avalon Project Documents in Law, History and Diplomacy" or "The BUE e-print repository" or "The Cupola: Scholarship at Gettysburg College" or "The European Jewish Research Archive" or "The IT University of Copenhagen's Repository" or "The Library of National Taiwan Normal Univ." or "The Management Univesity of Africa Repository" or "The National Museum of Western Art, Tokyo - Publications Repository" or "The Oberta in open access" or "The Open Repository @Binghamton (The ORB)" or "The Orange Grove" or "The Parthenon Frieze Repository" or "The Research Output Service" or "The Seoul Institute Repository" or "The UBM Repository" or "The University of Edinburgh Collections" or "The West Pomeranian Digital Library" or "Theoreme" or "Theses de l'ULP" or "Tiger Scholar Commons" or "Tokyo National Research Institute for Cultural Properties - Publications" or "TopSCHOLAR" or "Tottori University research result repository" or "Toulouse Capitole Publications" or "Trakya University Academic Open Access System" or "Traveling Culture: Circuit Chautauqua in the Twentieth Century" or "TREASURES @ UTD" or "TriCollege Digital Library" or "Trinity Digital Collections" or "Trinity's Access to Research Archive" or "Trisakti University Collection of Scholarly and Academic Pa" or "Tropmed Central Antwerp" or "TRUSpace" or "Tsukuba Repository" or "TU Delft Repository" or "tudigit" or "Tufts Digital Library" or "TUGraz OPEN Library" or "tukart" or "tuprints" or "TUT Digital Open Repository" or "TUT DPub" or "TWCU Repository" or "Tyler Collection of Romanian and Modern Art: University of Tasmania" or "Tz'ibal Naah" or "U-Now" or "u:scholar" or "UAJY repository" or "UAL Research Online" or "UB ScholarWorks" or "Ubi Thesis - Conhecimento Online" or "UCD Digital Library" or "UCL Discovery" or "UCLA Biblioteca de Medicina" or "UCrea" or "UCT Computer Science Research Document Archive" or "Udinus Repo" or "UDOspace" or "UEF Electronic Publications" or "UEF eRepository" or "uiana" or "UILSPACE" or "UIN Maliki Malang Repository" or "UiS Brage" or "UK Data Service ReShare" or "UKM Journal Article Repository" or "UKnowledge" or "ULB Sachsen-Anhalt HALCoRe" or "Ulsan College" or "Ulukau: The Hawaiian Electronic Library" or "UM Publications" or "Umm Al-Qura University Reference Repository" or "UMW Libraries Digital Collections" or "UNAM Scholary Repository" or "UNC Digital collections" or "UNEJ Repository" or "UNH Scholars' Repository" or "UNIB Scholar Repository" or "Unika Repository" or "UniKL IR" or "UnipiEprints" or "UniSA Research Outputs Repository" or "UnissResearch" or "Unitec Research Bank").mp.

22 ("United Nations Digital Library Islamabad" or "Unitn-eprints PhD" or "Univ. Duesseldorf: Duesseldorfer Dokumenten- und Publikationsserver" or "Univeristy of Warmia and Mazury Digital Library" or "Universidad Ricardo Palma" or "Universidade do Minho: RepositoriUM" or "Universitas Ahmad Dahlan Repository" or "Universitas Airlangga Repository" or "Universitas Islam Negeri Sultan Syarif Kasim Riau Repository" or "Universite Hassiba Benbouali Chlef -Algerie" or "Universiteit Twente Repository" or "Universiti Malaysia Kelantan Intitutional Repository" or

"University Archives" or "University Department of Health Studies Repository" or "University of Adelaide Library Electronic Text Collection" or "University of Arizona Campus Repository" or "University of Babylon Repository" or "University of Bath's research portal" or "University of Bedfordshire Repository" or "University of Birmingham Research Archive, E-papers Repository" or "University of Birmingham Research Archive, E-prints Repository" or "University of Birmingham Research Archive, E-theses Repository" or "University of Biskra repository" or "University of Biskra Theses Repository" or "University of Botswana Research, Innovation and Scholarship Archive" or "University of Brighton Repository" or "University of British Columbia's Information Repository" or "University of Chicago Library Digital Activities" or "University of Cincinnati Digital Resource Commons" or "University of Dar es Salaam" or "University of Debrecen Electronic Archive" or "University of Derby Online Research Archive" or "University of Dundee Online Publications" or "University of Florida Digital Collections" or "University of Florida Law Repository" or "University of Fukui Repository" or "University of Ghana Digital Collection" or "University of Groningen Research Database" or "University of Hertfordshire Research Archive" or "University of Huddersfield Repository" or "University of Hull Worktribe CRIS" or "University of Idaho Library Digital Initiatives" or "University of Innsbruck Digital Library" or "University of Leicester's OER Repository" or "University of Limpopo" or "University of Liverpool Repository" or "University of Malaya Students Repository" or "University of Mary Washington's Digital Library Repository" or "University of Maryland, Baltimore County" or "University of Miami Libraries Scholarly Repository" or "University of Michigan Library Repository" or "University of Minnesota Digital Conservancy" or "University of Minnesota IMAGES" or "University of Nahdlatul Ulama Surabaya Repository" or "University of Nevada, Las Vegas Repository" or "University of Nevada, Reno ScholarWorks Repository" or "University of New Orleans Digital Research Collections" or "University of Oregon Scholars' Bank" or "University of Oulu Repository - Jultika" or "University of Pretoria Electronic Theses and Dissertations" or "University of Queensland eSpace" or "University of Reading Research Data Archive" or "University of Regensburg Publication Server" or "University of Rwanda Repository" or "University of South Wales Research Explorer" or "University of Southern Denmark Research Output" or "University of St Andrews Research Portal" or "University of the Free State ETD" or "University of the Ryukyus Repository" or "University of Toyama Repository" or "University of Victoria Digital collections" or "University of Wales Trinity Saint David" or "University of Washington Structural Informatics Group Publications" or "University of West Bohemia Digital Library" or "University of Worcester Research and Publications" or "University of Yangon Repository" or "University of Zagreb Medical School Repository" or "University of Zambia Repository" or "University of Zululand Repository" or "univOAK" or "UNSWorks" or "UNT Digital Library" or "UNTHSC Scholarly Repository" or "UOC e-Repository" or "UPCommons. Portal del coneixement obert de la UPC" or "UPLACE" or "UPN Jatim Repository" or "UPSpace at the University of Pretoria" or "USC Digital Library" or "USC Research Bank - University of the Sunshine Coast" or "USFSP Digital Archive" or "USRA Houston Repository" or "UTC Digital Collections" or "UTC Scholar" or "UTL Repository" or "UTokyo Repository" or "Utsunomiya University Academic Information Repository" or "UUM Repository" or "UVH Repository" or "UVT e-doc" or "UWC Theses and Dissertations" or "UWISpace" or "UWL Repository" or "UWS Research Direct" or "Valley of the Shadow" or "ValpoScholar" or "Vanderbilt Electronic Thesis and Dissertation Archive" or "VCU Libraries Digital Collections" or "VCU Scholars Compass" or "VGTU repository" or "VID:Open" or "Virginia Henderson Global Nursing e-Repository" or "Virtual Archive of Polish Armenians" or "Virtual Commons - Bridgewater State University" or "Virtual Jamestown" or "Virtual Library of Cieplan" or "Virtual Library on Capacity Development" or "Virtual Reading Room of The John Paul II Catholic University of Lublin" or "Visual Arts Data Service" or "Vitela: Repositorios Institucional de la Pontificia Universidad Javeriana" or "VIUSpace" or "viXra" or "VizieR Catalogue Service" or

JMLA Journal of the Medical Library Association
SC
Supplemental content to
J Med Libr Assoc. Apr;108(2):dx.doi.org/10.5195/jmla.2020.856
www.jmla.mlanet.org
© Demetres, Delgado, Wright 2020

"VOCEDplus" or "Volltextserver der Universitaet Bamberg" or "Volltextserver der Virtuellen Fachbibliothek Psychologie" or "Vrije Universiteit Brussel Research Portal" or "VTechWorks" or "VTT Publications Register" or "VUT DigiResearch" or "Wageningen Staff Publications" or "Warsaw University of Technology Repository" or "Warwick Digital Library" or "Warwick Research Archives Portal Repository" or "Washington Research Library Consortium - Digital Collections" or "Washington Research Library Consortium Special Collections" or "Waterford Institute of Technology Repository" or "Web-based Archive of RIVM Publications" or "Wejherowo Digital Library" or "WesScholar" or "West DC" or "Western CEDAR" or "WestminsterResearch" or "White Rose E-theses Online" or "White Rose Research Online" or "Widya Mandala Catholic University Surabaya Repository" or "Wielkopolska Biblioteka Cyfrowa" or "Wikimedia Commons" or "William & Mary Law School Scholarship Repository" or "Wilson Center Digital Archive" or "Windesheim Repository" or "WinnSpace Repository" or "Wintec Research Archive" or "Wireless U" or "Wissenschaftliche Publikationsserver der Frankfurt University of Applied Sciences" or "WMSU" or "Wolverhampton Intellectual Repository and E-theses" or "Woods Hole Open Access Server" or "World Digital Library" or "WorldFish Center Publications" or "WSU Libraries Digital Collections" or "Xios Theses" or "Yale Law School Legal Scholarship Repository" or "Yale Medicine Thesis Digital Library" or "Yalova University Open Access Archive" or "Yamaguchi University Navigator for Open access Collection and Archives" or "Yanka Kupala State University of Grodno Publications (GRSU Publications)" or "York Digital Library" or "YorkSpace" or "Zaloamati" or "ZENODO" or "ZHAW digitalcollection" or "Zhytomyr State University Library" or "Zurich Open Repository and Archive").mp.

23 or/1-22
